# Robustness of close‐kin mark–recapture estimators to dispersal limitation and spatially varying sampling probabilities

**DOI:** 10.1002/ece3.6296

**Published:** 2020-05-05

**Authors:** Paul B. Conn, Mark V. Bravington, Shane Baylis, Jay M. Ver Hoef

**Affiliations:** ^1^ Marine Mammal Laboratory Alaska Fisheries Science Center NOAA National Marine Fisheries Service Seattle WA USA; ^2^ CSIRO Marine Lab Hobart TAS Australia

**Keywords:** abundance estimation, incomplete mixing, sampling bias, spatial heterogeneity

## Abstract

1. Close‐kin mark–recapture (CKMR) is a method for estimating abundance and vital rates from kinship relationships observed in genetic samples. CKMR inference only requires animals to be sampled once (e.g., lethally), potentially widening the scope of population‐level inference relative to traditional monitoring programs.
2. One assumption of CKMR is that, conditional on individual covariates like age, all animals have an equal probability of being sampled. However, if genetic data are collected opportunistically (e.g., via hunters or fishers), there is potential for spatial variation in sampling probability that can bias CKMR estimators, particularly when genetically related individuals stay in close proximity.
3. We used individual‐based simulation to investigate consequences of dispersal limitation and spatially biased sampling on performance of naive (nonspatial) CKMR estimators of abundance, fecundity, and adult survival. Population dynamics approximated that of a long‐lived mammal species subject to lethal sampling.
4. Naive CKMR abundance estimators were relatively unbiased when dispersal was unconstrained (i.e., complete mixing) or when sampling was random or subject to moderate levels of spatial variation. When dispersal was limited, extreme variation in spatial sampling probabilities negatively biased abundance estimates. Reproductive schedules and survival were well estimated, except for survival when adults could emigrate out of the sampled area. Incomplete mixing was readily detected using Kolmogorov–Smirnov tests.
5. Although CKMR appears promising for estimating abundance and vital rates with opportunistically collected genetic data, care is needed when dispersal limitation is coupled with spatially biased sampling. Fortunately, incomplete mixing is easily detected with adequate sample sizes. In principle, it is possible to devise and fit spatially explicit CKMR models to avoid bias under dispersal limitation, but development of such models necessitates additional complexity (and possibly additional data). We suggest using simulation studies to examine potential bias and precision of proposed modeling approaches prior to implementing a CKMR program.

## Graphical abstract

We examined the robustness of close‐kin mark–recapture (CKMR) estimators of abundance, survival, and recruitment under dispersal limitation and nonrandom sampling. We found that CKMR provides reasonably unbiased estimates when (a) dispersal is population‐wide or (b) when sampling is close to random. Abundance estimates are negatively biased when sampling is spatially restricted and there is dispersal limitation.

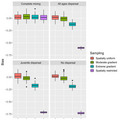

## 1 INTRODUCTION

Ecologists and natural resource professionals often require estimates of abundance and vital rates (e.g., fecundity, survival) to investigate population‐level processes and to manage fish and wildlife populations. Close‐kin mark–recapture (CKMR) is a recently developed technique for estimating abundance and demography of animal populations from the frequency of kinship relationships (e.g., parent–offspring, half‐siblings) observed in genetic samples (Bravington, Skaug, & Anderson, 2016; Skaug, 2001). In essence, offspring “mark” their parents and the frequency with which parents are encountered can be used to estimate adult abundance. Frequencies of half‐sibling pairs (HSPs) can also be used to estimate abundance, and provide additional information about adult survival since a parent must have survived from the older sibling’s birth date to the younger sibling's birth date in order to have reproduced (Bravington, Skaug, et al., 2016). Precision of estimates is considerably improved if ages can be estimated or inferred at the time of sampling (e.g., from age–length relationships). If data are rich enough, reproductive schedules can also be estimated.

As opposed to standard capture–recapture, which requires multiple encounters of the same animal, CKMR estimation can be conducted with animals that have only been encountered once. It is thus a potential “holy grail" for fish and wildlife agencies, who frequently have access to samples of harvested animals, often at a fraction of the cost of intensive marking and subsequent recapture operations necessary for traditional mark–recapture or mark–recovery modeling. Thus far, CKMR has been successfully applied to salmon (Rawding, Sharpe, & Blankenship, 2014), tuna (Bravington, Grewe, & Davies, 2016), shark (e.g., Hillary et al., 2018), and brook trout (Ruzzante et al., 2019) populations, but there is considerable interest in applying it to other marine, fresh water, and terrestrial animal populations.

According to Bravington, Skaug, et al. (2016), one requirement for CKMR estimation is that “…the event of an adult's being sampled should be independent of the number of its offspring sampled, conditional on covariates.” If sampling is spatially biased, and if parents and offspring are close together, this independence assumption is violated unless spatial location is explicitly modeled (see Discussion). For instance, many terrestrial mammals disperse a limited distance from their place of birth. If sampling is concentrated in a particular area or set of areas, the expected number of related animals in the sample may be higher than if animals were sampled with equal probability (hereafter, “random sampling”). If sampling is opportunistic, some level of spatial bias will often occur since fishers and hunters frequently concentrate their efforts in areas of high abundance or easy access (e.g., close to roads; Diefenbach et al., 2005). However, to our knowledge no one has investigated the degree of bias in CKMR estimators in such a situation. The closest example is by Davies, Bravington, and Thomson (2017), who suggested potential for considerable bias when applying naive CKMR estimators to Atlantic bluefin tuna populations. They concluded that unbiased estimation required adequate sampling in different spawning and nursery areas and use of CKMR models that explicitly account for spatial variation in stock structure.

Although it is certainly possible in principle to devise and fit spatially explicit CKMR models which should avoid bias (see Discussion), there is likely to be considerable additional modeling complexity entailed as well as additional data requirements. Thus, it may be preferable to fit the much simpler naive nonspatial CKMR models, even if there is a small price to pay in potential bias. Simulations can help determine in advance whether such bias is likely to be bad enough to justify the development of spatial models, collection of additional data, and/or to change the sampling scheme.

In the present paper, we investigate the robustness of CKMR estimators when dispersal is limited and there is spatial bias in sampling probabilities. In particular, we use spatially explicit, individual‐based simulation to record pedigrees and event histories (birth, death, location) under different movement and sampling scenarios applied to a long‐lived mammal population. We then investigate bias of CKMR estimators for abundance, survival, and relative fecundity at age that ignore spatial information. We also assess the power of goodness‐of‐fit tests to detect lack of mixing. The remainder of the paper is organized as follows. First, we provide a brief review of CKMR models and their basic assumptions. Second, we describe diagnostics to help ecologists detect dispersal limitation. Next, we describe our simulation study in further detail. After reporting results, we close with thoughts on applying nonspatial CKMR models to populations with dispersal limitation and spatially biased sampling.

## 2 MATERIALS AND METHODS

### 2.1 Notation and canonical CKMR models

Let *i* and *j* denote two individuals sampled from a population, and let $Y_{\mathrm{ijk}}$ be a binary random variable indicating whether animals i and j have a particular kinship relationship k (e.g., mother–offspring pair). For ease of exposition, we assume kinship relationships are determined with certainty—modifications will often be needed in real‐world applications, especially for half‐siblings. Under lethal sampling, we write the probability of a particular kinship relationship as $P[Y_{\mathrm{ijk}}=1|z_{i},z_{j}]$ to emphasize that the probability is conditional on covariate vectors $z_{i}$ and $z_{j}$ gathered for the two animals. Common covariates in canonical CKMR models include year of sampling ( $t_{i},t_{j}$ ) and year of birth ( $b_{i},b_{j}$ ) if ages can be obtained reliably; in many applications, ages are either imprecise or only a proxy such as length is available (e.g., for fish), but we ignore that complication here. In the following, we shall sometimes refer to $P[Y_{\mathrm{ijk}}=1|z_{i},z_{j}]$ as $P_{\mathrm{ijk}}$ for brevity. We follow the convention of Bravington, Skaug, et al. (2016) in using expected relative reproductive output (ERRO) to formulate expressions for $P_{\mathrm{ijk}}$ (for an alternate formulation, see Skaug, 2017). Here, ERRO is defined as the expected reproductive output of individual i relative to the population. For example, if k=1 denotes mother–offspring pair, the probability that a sampled female *i* is the mother of *j* can be written as

$$P_{ij1}=\frac{E[R_{i}\left(b_{j}\right)|z_{i},z_{j}]}{E[R_{+}\left(b_{j}\right)|z_{j}]},$$

where $E[R_{i}\left(b_{j}\right)|z_{i},z_{j}]$ is the expected reproductive output of female i in the year of *j*’s birth, and $E[R_{+}\left(b_{j}\right)]$ is the total expected reproductive output of the population in year $b_{j}$ (Bravington, Skaug, et al., 2016). Half‐sibling formulations for $P_{\mathrm{ijk}}$ are similar, though they necessarily involve the probability of a common parent producing offspring at $b_{i}$ and $b_{j}$ and surviving from $b_{i}\to b_{j}$ . Note that the formulae for computing ERRO can vary from simple to complicated depending on the biology and sampling: for example, on whether covariates such as age are measured accurately, and on whether sampling is lethal (Bravington, Skaug, et al., 2016).

Regardless of the specification for $P_{\mathrm{ijk}}$ , the ultimate goal of CKMR is to make inferences about abundance and demographic parameters given data on observed kinship relationships. The frequency of parent–offspring pairs (POPs) often provides information on abundance and fecundity, while HSP frequencies provide information about abundance and adult survival. Inference proceeds by maximizing the pseudolikelihood

(1) $$L=\prod_{i}\prod_{j}\prod_{k}P_{\mathrm{ijk}}^{y_{\mathrm{ijk}}}{\left(1-P_{\mathrm{ijk}}\right)}^{\left(1-y_{\mathrm{ijk}}\right)},$$

where $y_{\mathrm{ijk}}$ is an observed binary datum indicating whether animals *i* and *j* were a match for kin relationship *k*. Here, *L* is termed a “pseudolikelihood” because it treats events as independent when in fact there are dependencies; for example, an animal cannot have two mothers. However, pseudo‐maximum‐likelihood estimates are unbiased, and variance estimates are accurate when sample sizes are low relative to the size of the population (Bravington, Skaug, et al., 2016).

Inference can proceed by minimizing $-\log\left(L\right)$ directly or by first incorporating prior distributions (e.g., for survival or fecundity parameters) in the form of additional likelihood components. Computation is considerably more efficient if L is first factored to rely on sufficient statistics (i.e., by grouping animals with the same covariate values).

### 2.2 Diagnostics

It is apparent that there are two ways of achieving random kinship samples. One is through “complete mixing,” whereby animals move sufficiently so that the expected distance between related individuals is equal to that of randomly chosen individuals. In this case, it does not matter if sampling is spatially biased, and opportunistic sampling will work perfectly well for CKMR estimation. A second way of obtaining random kinship samples is through simple random sampling. For instance, if sampling effort is allocated uniformly throughout a species’ range, and each animal has an equal probability of being sampled, then it does not matter whether there is complete mixing. However, if animals do not mix thoroughly and sampling is spatially biased (e.g., through opportunistic sampling), animal abundance may be underestimated (Davies et al., 2017).

**FIGURE 1:**
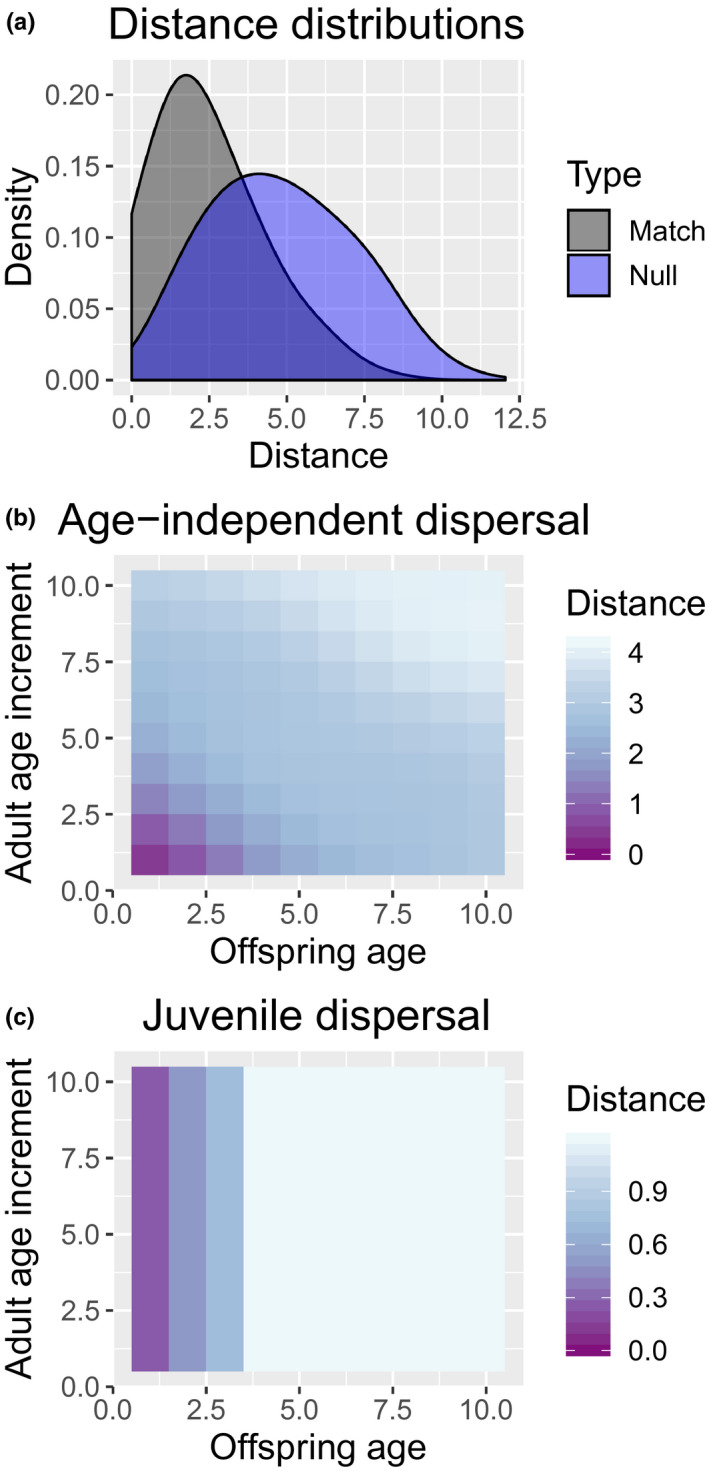
An example of potential information about dispersal that can be gained by examining distances between sampled kin pairs. In (a), the distribution of kin pair distances is shifted to the left from the null distribution of all possible comparisons of sampled animals, strongly suggesting incomplete mixing due to dispersal limitations. In (b), average distances among kin pairs were simulated for the case where both adults and juveniles exhibit diffusive dispersal, such that distances tend to increase with both offspring age and adult age increment (time since offspring birth). In (c), only juveniles were allowed to disperse, so that average distances increase initially for young animals, but do not change as a function of adult age

A variety of approaches can be used to assess the mixing assumption, including plotting telemetry data or examining the sampling locations of kinship matches relative to a null distribution of comparisons (Hillary et al., 2018). One simple diagnostic test is to compare histograms of observed distances (Figure 1a). If the distribution of distances for kinship matches is shifted to the left of a null distribution of distances between all pairs of animals, it is a good indication of lack of mixing. The mixing assumption can be formally tested as well, for example, using Kolmogorov–Smirnov tests (Hollander, Wolfe, & Chicken, 2013) (see Tests for incomplete mixing for an example). Note also that there is additional information about dispersal that can be gained by examining how distances between kin pairs change as a function of offspring age or adult age increment (i.e., time since offspring birth). In particular, observed patterns can be indicative of different types of dispersal (Figure 1b,c).

If diagnostic tests reveal that animals mix well, there is little reason to suspect that nonrandom sampling will bias CKMR estimators. However, if basic biology or diagnostic tests reveal a lack of mixing, what then? Must CKMR models account for movement in these cases to remain unbiased (presumably requiring additional data)? Or will CKMR models that ignore space suffice in some situations? Through simulation, we study these questions next.

### 2.3 Simulation study

#### 2.3.1 Data generating models

**FIGURE 2:**
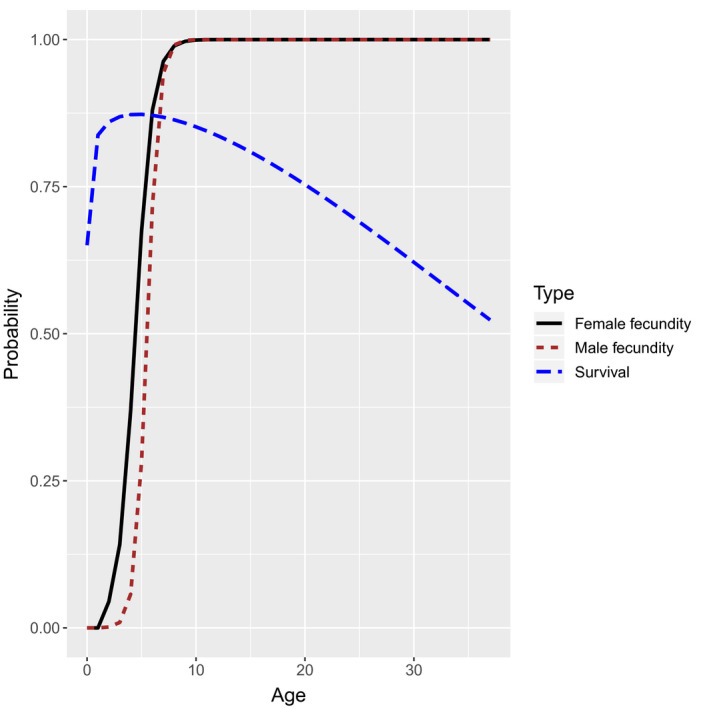
Age‐specific survival and reproductive schedules used to simulate CKMR data. Reproductive schedules were provided as a fixed input to the estimation models, whereas prior distributions on reduced additive Weibull parameters were provided for survival. CKMR, close‐kin mark–recapture

We conducted a simulation study to examine potential bias in CKMR estimators when estimating abundance in dispersal‐limited populations. Our data generating and estimation models are built loosely on the life history of bearded seals in Alaska (a population we are interested in applying CKMR methods to), but should be somewhat typical of long‐lived mammals subject to a relatively low rate of exploitation. Our simulations used mortality‐at‐age estimates derived from hierarchical analysis (Trukhanova, Conn, & Boveng, 2018), together with logistic fecundity‐at‐age estimates based on data from reproductive schedules (London, 2019) (Figure 2). Annual survival probability at age (*a*) followed a 3‐parameter reduced additive Weibull function (RAW; Choquet, Viiallefont, Rouan, Gaanoun, & Gaillard, 2011):

$$S_{a}=\exp\left(-{\left(\eta_{1}a\right)}^{\eta_{2}}-{\left(\eta_{1}a\right)}^{1/\eta_{2}}-\eta_{3}a\right).$$

We increased mortality from the original bearded seal estimates of Trukhanova et al. (2018) until the expected finite population growth rate (Caswell, 2001) was approximately 1.0 to ensure a stable population (the schedule used in simulations appear in Figure 2). For fecundity at age, we used logistic models for each sex (*g*), parameterized as.

$$f_{a,g}={\left[1+\exp\left(-\nu_{g,1}*\left(a-\nu_{g,2}\right)\right)\right]}^{-1},$$

where the parameters $\nu_{g,i}$ were chosen to match bearded seal fecundity‐at‐age estimates from London (2019). Our motivation for using parametric models here was to be able to estimate a manageable number of survival and fecundity parameters when analyzing simulated CKMR data. The exact values used are provided in Appendix S1.

We simulated population dynamics on a 10 × 10 grid using an individual‐based modeling approach where parents could only mate with individuals in their own cell. Starting with a population size of 10,000 distributed randomly across the grid, we simulated data for 60 years. Our model employed a postbreeding census (sensu Caswell, 2001), with mortality, movement, and breeding implemented sequentially. Mortality was simulated via independent, uniform draws with probability determined according to the curve in Figure 2. Movement was determined by a random draw, with $\psi_{a}^{m,n}$ , the probability of an age a animal moving from cell m to cell n , determined according to one of three simulation scenarios (see below). Mating was simulated by first determining whether a female breeds, with probability given in Figure 2. For females that breed, their mate was determined by selecting an available male in the same cell with probability proportional to age‐specific male fecundity values (Figure 2). Breeding females produced one offspring per year.

We considered four dispersal scenarios, corresponding to age‐independent dispersal, juvenile dispersal, no dispersal, or completely random movement (such that location in one year is independent of location in the previous year). The age‐independent dispersal scenario corresponded to the situation where movement probabilities were constructed with a Gaussian kernel:

$$\psi_{a}^{m,n}\propto N\left(d\left(m,n\right);0,1\right),$$

where $N\left(d\left(m,n\right);0,1\right)$ gives a standard normal probability density function evaluated at $d\left(m,n\right)$ , the distance between the centroids of grid cells m and n (grid cells were defined to have length and width = 1.0). The juvenile dispersal scenario used the same kernel for a=0 , with $\psi_{a}^{m,n}=0$ for a>0 .

**FIGURE 3:**
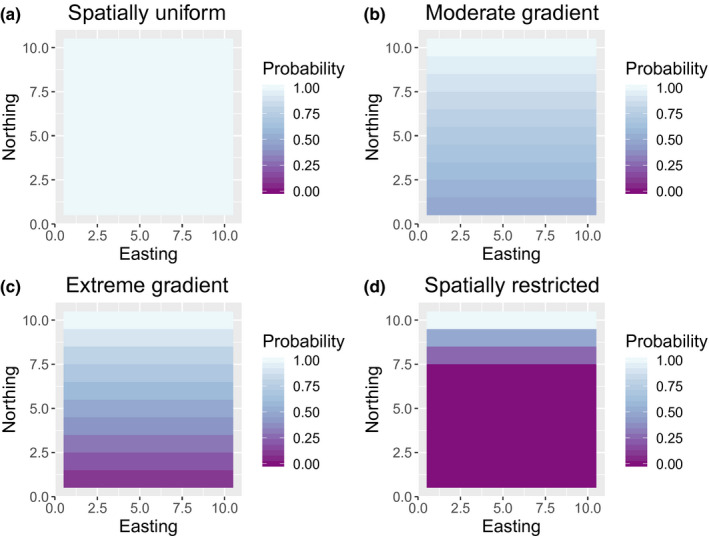
Different sampling scenarios for simulation study, corresponding to spatially uniform sampling (a), a moderate gradient in sampling probabilities (b), an extreme gradient in sampling probabilities (c), and spatially restricted sampling, where sampling only occurs near the north edge of the study area (d). Values represent the relative probability of sampling an individual in a given cell compared to the cell(s) with the highest probability

We implemented four sampling scenarios corresponding to random sampling, sampling on a moderate gradient, sampling on an extreme gradient, and sampling on the northern end of the study area only (Figure 3). The moderate gradient scenario was configured so that the probability of sampling an individual on the northern end of the study area was twice as high as on the southern side, with a smooth transition in between; in the extreme gradient scenario, there was a 10‐fold variation in sampling probability. Simulations were initialized using a stable age distribution and assuming virtual animals were uniformly distributed among grid cells. We ran simulations for 60 years, with sampling configured to occur over the last 20 years of each time series, with n=100 newly dead animals sampled per year, mimicking the situation where genetic samples are obtained from hunter‐ or angler‐killed animals. The duration and intensity of sampling were selected to be roughly similar to those available from indigenously harvested bearded seals in Alaska (B. Taras and L. Quakenbush, Alaska Dept. of Fish & Game, unpublished data). We selected the 60‐year simulation time frame to eliminate “founder effects”—virtual animals initialized at the beginning of the study had a negligible probability of surviving to the period when sampling began. For each simulation scenario, we simulated 100 data sets.

#### 2.3.2 Estimation

We analyzed each simulated CKMR data set with a naive CKMR model (i.e., assuming no spatial structure). We simultaneously modeled mother–offspring, father–offspring, maternal half‐sibling, and paternal half‐sibling pairs within a joint likelihood. We did not model full‐sibling pairs as the frequency of these occurring in nature is often quite low in mating systems without pair bonding. To calculate the probability of each kinship relationship, we embedded a deterministic population dynamics model into the pseudolikelihood. We attempted to estimate abundance, age‐specific survival, and fecundity‐at‐age, with informative prior distributions on survival and fecundity parameters (see below).

To formalize the population dynamics model, let $N_{a,t,g}$ gives the expected number of animals that are age a ( $a\in \left\{0,1,\cdots ,37\right\}$ ) and sex g ( g=1 for females and g=2 for males) in year t , $t\in \left\{1,2,\cdots ,60\right\}$ . Assuming new recruits are 50% female, we set $N_{0,1,g}=\exp\left(R_{0}\right)$ , where $R_{0}$ is an estimated parameter. We assume a stable age structure at beginning of the time series with $N_{a+1,1,g}=N_{a,1,g}S_{a}$ , $S_{a}$ being survival of age class a (assumed here to be independent of sex and year). For later years, survival was modeled as $N_{a+1,t,g}=N_{a,t-1,g}S_{a}$ for 2≤t≤50 and 0≤a≤36 . The probability of surviving past age 37 was negligible, so we did not model later age classes. Recruitment for t>0 was modeled by applying the female‐specific fecundity‐at‐age vector ( $f_{a,1}$ ) from Figure 2, specifically $N_{0,t,g}=0.5\sum_{a}N_{a,t,1}f_{a,1}$ .

Given that we used a postbreeding census, we calculated the probability of POPs as the ratio of the prospective parent's expected reproductive output in the year prior to the offspring's birth relative to the total reproductive output then. To speed computation, we found it useful to summarize kinship probabilities according to sufficient statistics. Defining $P_{b_{1},y_{1},b_{2},g}$ to be the probability of a POP for comparisons that have parent birth year $b_{1}$ , parent capture year $y_{1}$ , offspring birth year $b_{2}$ , and parent sex g , we have

$$P_{b_{1},y_{1},b_{2},g}=\left\{\begin{array}{cc} \frac{f_{b_{2}-b_{1}-1,g}}{\sum_{a}N_{a,b_{2},g}f_{a-1,g}} & \text{if}\;y_{1}\geq b_{2},b_{1}<y_{1}\leq \left(b_{1}+37\right),\;\text{and}\;b_{2}\leq \left(b_{1}+37\right)\\ 0 & \text{otherwise} \end{array}\right\}$$

Probabilities of HSPs are slightly more complicated, as we must integrate (sum) over possible ages of the parent. In this case, we summarize probabilities as $P_{b_{1},b_{2},g}^{{}^{\prime }}$ , where $b_{1}$ and $b_{2}$ in this case are birth years of the older and younger sibling, respectively:

(2) $$P_{b_{1},b_{2},g}^{\prime }=\sum_{a}N_{a,b_{1},g}\frac{f_{a-1,g}}{\sum_{a^{\prime }}N_{a\prime ,b_{1},g}f_{a\prime -1,g}}\left(\prod_{y=b_{1}}^{b_{2}-1}S_{a+y-b_{1}}\right)\frac{f_{a+b_{2}-b_{1}-1,g}}{\sum_{a\prime }N_{a\prime ,b_{2},g}f_{a\prime -1,g}}$$

We assume that the sex ( g ) of the shared parent in half‐sibling matches is known, as can often be determined if there is sufficient mitochondrial haplotype diversity. Note that an equivalent form of equation (2) also appears in Hillary et al. (2018) (Online supplement, page 19).

Using a Poisson approximation to the binomial distribution, we write the negative joint log pseudolikelihood for the naive CKMR model as

$$\begin{array}{c} \Lambda =\sum_{b1}\sum_{y1}\sum_{b2}\sum_{g}m_{b_{1},y_{1},b_{2},g}\log\left(n_{b_{1},y_{1},b_{2},g}P_{b_{1},y_{1},b_{2},g}\right)-n_{b_{1},y_{1},b_{2},g}P_{b_{1},y_{1},b_{2},g}+\\ \sum_{b_{1}}\sum_{b_{2}}\sum_{g}m_{b_{1},b_{2},g}^{\prime }\log\left(n_{b_{1},b_{2},g}^{\prime }P_{b_{1},b_{2},g}^{\prime }\right)-n_{b_{1},b_{2},g}^{\prime }P_{b_{1},b_{2},g}^{\prime }-\\ \sum_{i=1}^{3}\log\left(N\left({\tilde{\eta }}_{i};\eta_{i},\sigma_{i}^{2}\right)\right)-\sum_{i=1}^{2}\sum_{g=1}^{2}\log\left(N\left({\tilde{\nu }}_{g,i};\nu_{g,i},\tau_{g,i}^{2}\right)\right) \end{array}$$

Here, n gives the number of pairwise comparisons made of a given type from the sampled set of individuals, and m gives the number of matches of a particular type. Symbols with a prime denote HSP comparisons, while those without denote POP comparisons. Note that we omitted POP comparisons for the case where the death year of the parent occurred in the year of a potential offspring's birth, as well as HSP comparisons for the case where individuals are the same age. These restrictions can be important in real‐world applications to prevent bias arising from dependent fates.

We also include Gaussian prior distributions on reduced additive Weibull (RAW) survival parameters and logistic fecundity parameters. For survival, $N\left({\tilde{\eta }}_{i};\eta_{i},\sigma_{i}^{2}\right)$ denotes a Gaussian prior on the i th log‐scale RAW parameter, ${\tilde{\eta }}_{i}$ , with mean $\eta_{i}$ and standard deviation $\sigma_{i}$ . Here $\eta_{i}$ was set to the values used to generate data, and $\sigma_{i}$ values were determined subjectively by plotting RAW curves with different parameter values until a RAW model with $\eta_{i}\pm 2\sigma_{i}$ appeared implausible (see Appendix S1 for values used). Similarly, for fecundity parameters, $N\left({\tilde{\nu }}_{g,i};\nu_{g,i},\tau_{g,i}^{2}\right)$ denotes a Gaussian prior on the i th logistic parameter for gender g . In this case, the mean $\nu_{g,i}$ was set to the data generating values and $\tau_{g,i}$ was set such to $0.4\nu_{g,i}$ (corresponding to a CV of 0.4).

For each scenario, we calculated mean proportional relative bias of abundance, and plotted true and estimated survival and fecundity schedules. Bias of the CKMR abundance estimator was summarized by averaging true and estimated abundance over the final 20 years of each simulation (the years of sampling). To compute true and estimated abundance, we summed totals of subadults and adults (ages 2+). In general, CKMR does not provide information about abundance of reproductively immature animals, although in our model the structure of the assumed population model allows predictions of younger age classes.

#### 2.3.3 Tests for incomplete mixing

To examine the ability of Kolmogorov–Smirnov tests to diagnose incomplete mixing, we ran additional simulations. Populations were simulated as in the previous section with the same combination of sampling and dispersal scenarios. However, we varied the intensity of sampling, examining cases where 15, 25, or 100 genetic samples were obtained per year (we term these “low”, “medium,” and “high” sampling intensities, respectively). These levels correspond to roughly 0.15%, 0.25%, and 1.0% of the population. For each case, we examined whether Kolmogorov–Smirnov tests could diagnose differences in the distribution of distances between HSPs and POPs from a null distribution of all possible comparisons. Since dispersal was configured to occur between years, we removed comparisons of distances that occurred (a) in the same year and (b) in the year of birth for one of the individuals.

#### 2.3.4 Computing

We simulated CKMR data in the R programming environment (R Development Core Team, 2017), using the fishsim package (Baylis, 2019) to simulate population dynamics, writing additional R functions to implement different movement scenarios and sample individuals (assumed to be dead from harvest). Estimation was performed by minimizing joint negative log pseudolikelihoods with the nlminb function with respect to the parameter vector θ . Estimated parameters consisted of $\theta =\left\{R_{0},{\tilde{\eta }}_{1},{\tilde{\eta }}_{2},{\tilde{\eta }}_{3},{\tilde{\nu }}_{1,1},{\tilde{\nu }}_{1,2},{\tilde{\nu }}_{2,1},{\tilde{\nu }}_{2,2}\right\}$ . Abundance and adult survival could be computed as functions of estimated parameters given the population dynamics model. We coded the log‐likelihood in C++ and linked it to R via the ADT package (available at https://github.com/pjumppanen/ADT) which allows automatic differentiation from Tapenade libraries (Hascoet & Pascual, 2013). The C++ and R code have been permanently archived in a publicly available repository (Conn, 2020).

## 3 RESULTS

**FIGURE 4:**
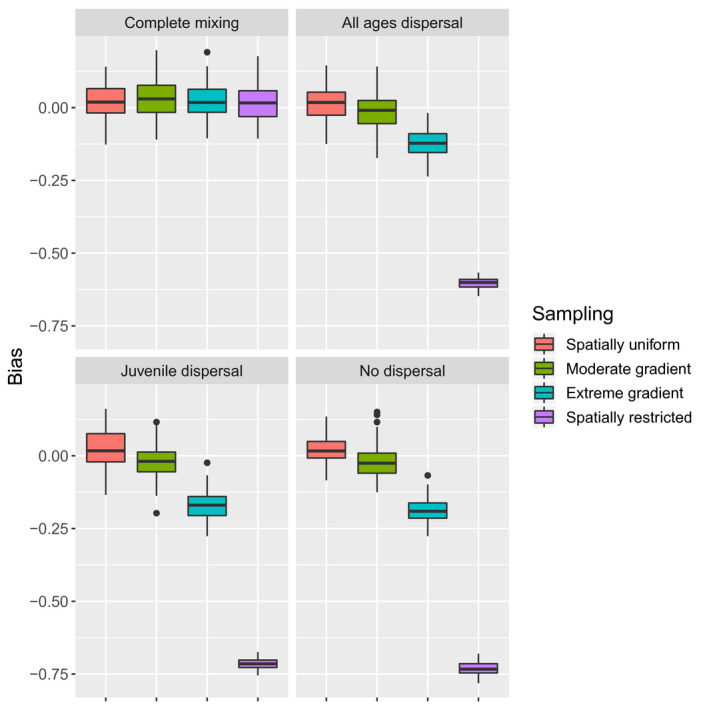
Proportional bias in abundance estimates as a function of dispersal type and sampling scenario. Possible dispersal types included complete mixing, age‐independent dispersal, juvenile‐biased dispersal, and no dispersal. Sampling scenarios included random sampling of dead animals irrespective of location (“Spatially uniform"), moderate or extreme gradients in sampling, or sampling that was restricted to the northern edge of each habitat grid (“Spatially restricted”). Each boxplot summarizes proportional bias in estimated abundance over 100 simulated data sets. Little to no bias was realized under complete mixing or under spatially uniform or moderate gradients in sampling probability. However, abundance was biased low under dispersal limitation when either an extreme gradient or spatially restricted sampling was simulated

For abundance, proportional relative bias was approximately 2% for scenarios where model assumptions were met (either complete mixing or constant sampling probabilities; Figure 4). For incomplete mixing (dispersal limitation) under a moderate sampling gradient, abundance was negatively biased by 1%–2% depending on scenario. The extreme sampling gradient and spatially restricted sampling scenarios were considerably negatively biased under dispersal limitation (12%–19% and 60%–73%, respectively). In general, greater mixing resulted in less bias for dispersal‐limited scenarios (Figure 4).

**FIGURE 5:**
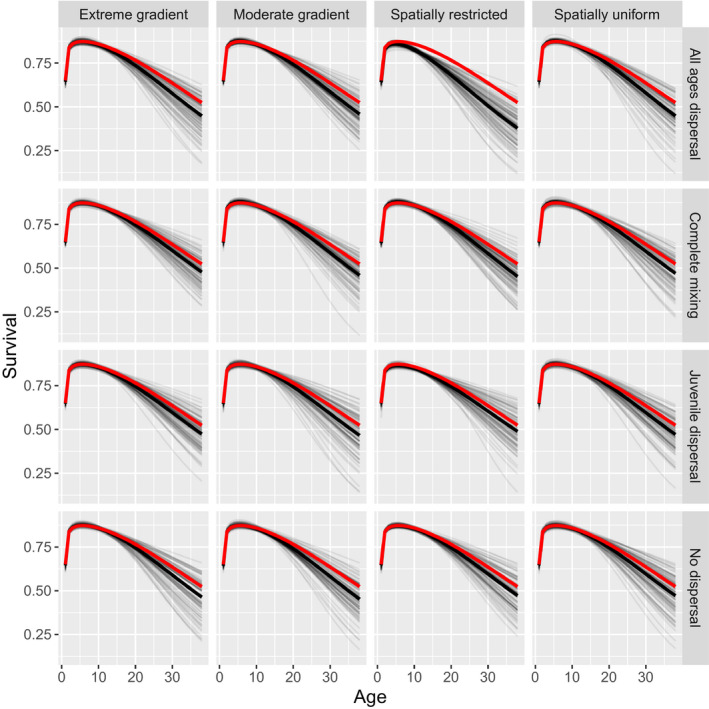
Age‐specific survival estimates for CKMR simulations by sampling (columns) and dispersal (rows) scenario. Thin gray lines represent estimates from individual simulation runs, while thick black lines represent means of all simulation replicates. Thick red lines represent values used to simulate data. If survival is recaptured perfectly, the dark red and dark black lines should overlap completely. Survival appears to be underestimated in older age classes in all scenarios, but at ages where there were few data (<2% of populations were over age 20). More concerning was when spatially restricted sampling was employed and adults dispersed out of the sampled populations (“All ages dispersal”). Here, adult survival is considerably underestimated, presumably because of permanent emigration

Survival was reasonably unbiased at younger ages (<15) for all scenarios except for one (Figure 5). When sampling was spatially restricted and dispersal was age‐independent, cumulative survival from ages 4 to 10 was underestimated by 15%. Presumably, this is because adults can emigrate away from the sampled area, and the CKMR model is unable to differentiate such emigration from mortality. Interestingly, there appeared to be a negative bias in survival at older age for all scenarios (e.g., >20), though there are admittedly few individuals reaching this age range (approximately 2% of the population). Ratios of Hessian‐based posterior standard errors to standard deviations of Bayesian prior distributions provide one indication of whether CKMR data provide information about parameters relative to model inputs. Ratios substantially less than 1.0 suggest that kinship data provide increased inference relative to prior assumptions, while ratios near 1.0 suggest that prior distributions are driving inferences. For survival, reduced additive Weibull survival parameters ( ${\tilde{\eta }}_{1},{\tilde{\eta }}_{2},{\tilde{\eta }}_{3}$ ) were all less than 1.0 (0.47, 0.81, and 0.37, respectively), indicating that CKMR data aided in their estimation. Note, however, that CKMR provides no information about survival of animals before they reach reproductive maturity; estimates for these ages are entirely a function of prior distributions and the assumed functional form of the survival curve.

**FIGURE 6:**
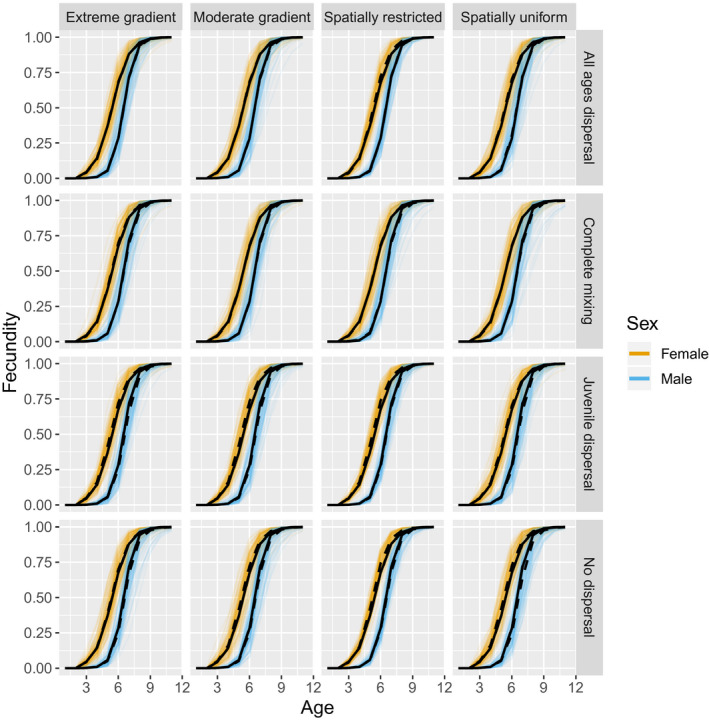
Age‐specific fecundity estimates for CKMR simulations by sampling (columns) and dispersal (rows) scenario. Thin orange and thin blue lines represent estimates from individual simulation runs for females and males, respectively, while dashed black lines are fecundity estimates averaged over all simulation replicates. Solid black lines represent values used to simulate data. Reproductive schedules were all unbiasedly estimated regardless of simulation scenario. CKMR, close‐kin mark–recapture

Reproductive schedules were all unbiasedly estimated (Figure 6) regardless of simulation scenario. Ratios of Hessian‐based posterior standard errors to prior standard deviations of logistic fecundity parameters ( ${\tilde{\nu }}_{1,1},{\tilde{\nu }}_{1,2},{\tilde{\nu }}_{2,1},{\tilde{\nu }}_{2,2}$ ) were 0.79, 0.83, 1.17, and 0.76. These ratios are relatively close to 1.0, suggesting estimation of fecundity parameters may rely more heavily on prior distributions than survival parameters, especially for the slope of the male fecundity curve.

**FIGURE 7:**
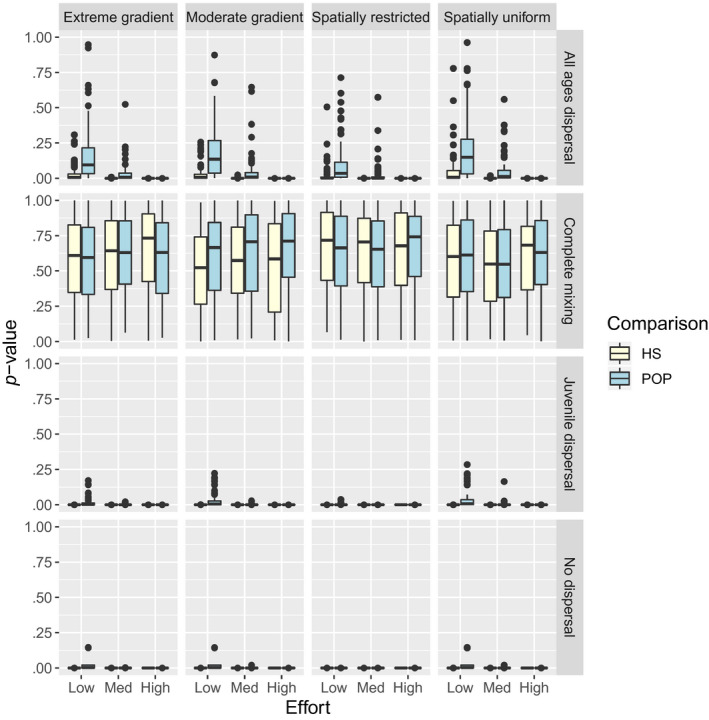
Boxplots summarizing the distribution of p‐values in additional simulations conducted to examine the ability of Kolmogorov–Smirnov tests to diagnose lack of mixing (i.e., dispersal limitation) using distances of kin pairs relative to null distributions of all possible comparisons. Simulations often resulted in low *p*‐values under dispersal limitation, suggesting that Kolmogorov–Smirnov tests had high power to discern dispersal limitation even in scenarios with “low” sampling effort (i.e., ≈0.15\% of the population was sampled)

Kolmogorov–Smirnov tests had high power to discriminate nonmixing of related individuals (Figure 7) for all cases except for POPs under low sampling intensity. For reference, the mean number of POPs for low, medium, and high sampling intensities was 5, 13, and 204, respectively. The mean number of HSPs for these scenarios was 21, 58, and 921.

## 4 DISCUSSION

In this paper, we examined whether CKMR can be an effective strategy for estimating animal abundance and demographic parameters when there is incomplete mixing of animals and sampling is spatially biased. When combined, these two conditions can potentially bias CKMR estimators because they can alter the frequency of kin‐pair matches from what one would expect in a randomly sampled population. Under the range of conditions simulated here, we have shown that abundance estimators from CKMR are reasonably robust to moderate variation in sampling effort under dispersal limitation, such as when effort is along a gradient varying by a factor of two. However, care should be taken when effort varies more dramatically (e.g., along a gradient with 10‐fold variation in sampling probabilities) or when there are areas that are unsampled. Survival and fecundity were more robust, with fecundity schedules reasonably estimated in all scenarios and adult survival only appreciably biased when it was possible for adults to emigrate out of the sampled area. As we might expect, CKMR only appears capable of estimating “apparent survival,” as with Cormack–Jolly–Seber models applied to conventional mark–recapture data (Williams, Nichols, & Conroy, 2002).

Fortunately, Kolmogorov–Smirnov tests reliably detected incomplete mixing even with relatively low sample sizes (e.g., with 25 animals genotyped per year; 0.25% of the population). Note, however, that the sample size required to get a sufficient number of kin pairs scales with the square root of abundance, so that increased sampling will be necessary to achieve similar power in larger populations than the 10,000 animals considered here. Even then, low p‐values do not by themselves indicate the likely level of bias. If sampling is spatially uniform, for instance, bias is still negligible; if sampling is opportunistic and concentrated in certain areas, bias may be considerable (Figure 4). If incomplete mixing is detected, we suggest that researchers conduct simulations tailored to their populations' dispersal and sampling dynamics to investigate possible levels of bias, and if necessary, construct spatially explicit CKMR models for estimation (see below). Another approach (suggested by a reviewer) would be to conduct diagnostics based on different age increments, with the hope that spatial dependence decreases with time since the year of birth (as in Figure 1). If such a relationship is found, inference could proceed using HSP or POPs where comparisons are limited to longer age increments. Although this approach would require higher sample sizes (both to detect incomplete mixing and to achieve adequate precision), it may be a way to reduce bias.

In this study, we limited consideration of models to those that were reasonably simple. This was for clarity, for ease of programming, and to limit computation time. Practical models for real populations will often need to address complications such as genotyping error and ageing error, which necessitate more intricate mathematics (Bravington, Skaug, et al., 2016). For instance, we assumed that kinship relationships were known perfectly. This is often a reasonable assumption for POPs, but more complex models will often be needed for HSPs because of false positives and because parental‐ and maternal‐half‐siblings may be difficult to discriminate in populations with low mitochondrial haplotype diversity. We suspect that biases for more sophisticated models will be similar to those estimated here, though precision will be reduced due to reduced sample sizes and/or an increased number of parameters.

As with all simulation studies, care should be taken not to extrapolate our findings to systems that are markedly different from the ones that we implemented here. Our simulations were geared toward long‐lived mammals with a relatively low rate of exploitation. Consequences of spatial structure on estimator bias are likely to vary from case to case. For instance, Davies et al. (2017) investigated consequences of ignoring spatial structure when sampling Atlantic bluefin tuna populations that were structured into several stocks with different spawning and nursery grounds. In their case, they found that estimates of abundance could be severely biased if spatial structure was not explicitly accounted for within CKMR models. It thus seems prudent to conduct CKMR scoping studies that are specifically tailored to the biology and sampling specifics of the individual population being investigated. Fortunately, tools we have described here (e.g., individual‐based simulation) should aid in exploring assumption violations.

In addition to simulation testing of naive CKMR models, it may be worth developing spatially structured CKMR models that explicitly allow movement. This might be particularly useful for populations where there is low mixing (which we have shown can be readily detected with reasonable sample sizes) coupled with strong spatial bias in sampling: for example, if samples are obtained by hunters or fishers that target areas preferentially owing to ease of access or perceived animal density. Such models would include spatial location of capture as an additional covariate, integrating over possible birth locations (Bravington, Skaug, et al., 2016, section 3.1.5) . However, such models would also need to account for spatial structure in relative reproductive output, which would in turn require a model for how abundance varies over space. It could be difficult to fit such a model to CKMR data alone when the observed number of kinship pairs is small (e.g.,<50) . In such cases, auxiliary data may be needed. For instance, coupling CKMR with a spatially explicit relative abundance index or with utilization distributions estimated from telemetered animals could provide the information needed to make estimation effective. Such models will also likely require extra assumptions, such as spatial homogeneity in population trend and temporal homogeneity in detection probability. However, there is clearly information in kinship patterns that can be exploited to estimate movement and migration rates (Bode, Williamson, Harrison, Outram, & Jones, 2018; Wang, 2014), information that could potentially be used in future CKMR models.

## 5 CONCLUSION

Close‐kin mark–recapture is in its infancy, and like traditional mark–recapture we foresee a radiating period of growth where models are developed to overcome obstacles, software is made more user‐friendly for ecologists, and ultimately CKMR is applied to monitor and manage a greater number of species. As enthusiastic as we are about its strengths, we think it important for ecologists to also understand its limitations. Incomplete mixing coupled with spatially biased sampling is certainly an important case, but there are a large number of other assumption violations (e.g., nonindependence of fates, alternate mating systems, heterogeneity in detection, trait‐based harvesting) that will need to be examined on a case‐by‐case basis.

## CONFLICT OF INTEREST

None declared.

## AUTHOR CONTRIBUTION

**Paul Conn:** Conceptualization (lead); Formal analysis (lead); Investigation (lead); Methodology (lead); Software (lead); Visualization (lead); Writing‐original draft (lead); and Writing‐review & editing (lead). **Mark Bravington:** Conceptualization (supporting); Formal analysis (supporting); Methodology (supporting); Software (supporting); and Writing‐review & editing (supporting). **Shane Baylis:** Methodology (supporting); Software (supporting); and Writing‐review & editing (supporting). **Jay Ver Hoef:** Conceptualization (supporting); Funding acquisition (lead); Resources (supporting); and Writing‐review & editing (supporting). 

## Supporting information

Appendix S1

## Data Availability

C++ and R code have been permanently archived at http://doi.org/10.5281/zenodo.3715496.
