# Supplementary material for: Robustness of close‐kin mark–recapture estimators to dispersal limitation and spatially varying sampling probabilities
Source: Ecol Evol. 2020 May 5;10(12):5558–69. doi: 10.1002/ece3.6296 (PMC7319163; doi:10.1002/ece3.6296)
Supplement: Supplementary file 1 — Appendix S1 [file ECE3-10-5558-s001.pdf]

Robustness of close-kin mark-recapture estimators to dispersal limitation and spatially varying sampling probabilitiesa

Paul B. Conn[1,*], , Mark V. Bravington[2], Shane Baylis[2], Jay M. Ver Hoef[1]

[1] Marine Mammal Laboratory, Alaska Fisheries Science Center, NOAA, National Marine Fisheries Service, 7600 Sand Point Way NE, Seattle, WA 98115 USA; [2] CSIRO Marine Lab, GPO Box 1538, Hobart 7001, TAS, Australia

*paul.conn@noaa.gov

Appendix S1: Close-kin mark-recapture simulation details

In this appendix, we provide more detail about how populations were simulated.

Spatial population dynamics model

Let $N_{a,t,g,s}$ give the number of age a and sex g animals that are in location s at time t. Here, location s refers to one of 100 grid cells on a (10 $\times$ 10) grid. We initialize the expected abundance in the first year of the model as a function of an unknown parameter R_0 and a spatial index I_s. In particular, we set $N_{0,1,g,s} = 0.5\exp(R_0)I_s$. For this paper, we assume the spatial index is constant with $I_s = 0.01$ (i.e. uniform expected abundance over the landscape). As with the naive model, a stable age distribution is assumed in each cell, so that $N_{a+1,1,g,s} = N_{a,1,g,s}S_{a,g}$ for $a > 0$ (here S_a gives survival of an age a individual). Following initialization, future dynamics proceed according to an individual based model. For each year, individuals first survive with probability S_a, possibly transition into a new spatial location s' with probability $\psi_a^{s,s'}$, and then possibly breed (see below).

We based survival on a reduced additive Weibull mortality model (Choquet *et al.* 2011), a formulation that allows for increasing survival with age, followed by senescence:

$$S_{a,t,g,s} = \exp\left(-c((\eta_1 a)^{\eta_2} + (\eta_1 a)^{1/\eta_2} + \eta_3 a)\right).$$

We used values of $\eta_1 = 0.055$, $\eta_2 = 2.80$, and $\eta_3 = 0.076$ based on predictions for bearded seals made in a hierarchical analysis of phocid seals (Trukhanova *et al.* 2018). We also incorporated a fixed constant $c = 1.3$ into the model to make the associated Leslie matrix (Caswell 2001) for the population have eigenvalues near 1.0 (so that population size was stable over time in each simulation).

In scenarios allowing movement, the probability that an animal was in spatial location s' at time $t+1$, given that it was in location s at time t and survived the interval was given by $\psi_a^{s,s'}$. When movement was completely random, $\psi_a^{s,s'} = 0.01$ for all s and s'. When movement was restricted, we set

$$\psi_a^{s,s'} \propto \text{Normal}(d(s,s'); 0, 1),$$

where Normal$(d(s,s'); 0, 1)$ is the standard normal probability density function evaluated at $d(s,s')$, the distance between the centroids of grid cell s and s'.

We implemented reproduction as follows. First, each female in the population was determined to breed with probability

$$f_{a,1} = ((1 + \exp(-1.264 * (a - 5.424))))^{-1}.$$

Here, values are representative of observed bearded seal sexual maturity. If females successfully bred, we determined the associated male by randomly sampling with replacement from the pool of males located in the same cell as the potential female, with probability proportional to

$$f_{a,2} = ((1 + \exp(-1.868 * (a - 6.5))))^{-1}.$$

© 2015 The Authors. Journal compilation © 2015 British Ecological Society, *Methods in Ecology & Evolution*
Prepared using besauth.cls

That is, females would be more likely to breed with older, fully reproductively mature males. Each breeding female gave birth to one offspring, the sex of which was determined randomly with a probability of 0.5.

This annual sequence of population dynamic steps was repeated for 60 years, with newly dead animals sampled in the final 20 years of simulation. The 40 year "burn-in" was necessary to prevent sampling founders (virtual animals alive at the time of simulation initialization) from being sampled, as these animals do not have any recorded parents. For all progeny, we kept track of parents to reconstruct kinship relationships necessary for CKMR estimation.

References

Caswell, H. (2001) *Matrix Population Models, 2nd Edition*. Sinauer, Sunderland, MA.

Choquet, R., Viallefont, A., Rouan, L., Gaanoun, K. & Gaillard, J.M. (2011) A semi-markov model to assess reliably survival patterns from birth to death in free-ranging populations. *Methods in Ecology and Evolution*, **2**(4), 383–389.

Trukhanova, I.S., Conn, P.B. & Boveng, P.L. (2018) Taxonomy-based hierarchical analysis of natural mortality: polar and subpolar phocid seals. *Ecology and evolution*, **8**(21), 10530–10541.

© 2015 The Authors. Journal compilation © 2015 British Ecological Society, *Methods in Ecology & Evolution*
